# Dissociation between humoral and multi-omic immune responses to inactivated COVID-19 vaccination in patients with pulmonary tuberculosis

**DOI:** 10.3389/fimmu.2026.1933038

**Published:** 2026-09-18

**Authors:** Zhijie Jiang, Pengfei Jin, Xiaorui Nie, Jin Wei, Siyue Jia, Qiao Liu, Mingwei Wei, Xiaoyan Ding, Daming Zhou, Liguo Zhu, Limei Zhu, Mulong Du, Jingxin Li

**Affiliations:** 1 School of Public Health, Southeast University, Nanjing, China; 2 Jiangsu Provincial Medical Innovation Center, National Health Commission Key Laboratory of Enteric Pathogenic Microbiology, Jiangsu Provincial Center for Disease Control and Prevention (Jiangsu Provincial Academy of Preventive Medicine), Nanjing, China; 3 Department of Biostatistics, Key Laboratory of Public Health Safety and Emergency Prevention and Control Technology of Higher Education Institutions in Jiangsu Province, Center for Global Health, School of Public Health, Nanjing Medical University, Nanjing, China; 4 The Affiliated Taizhou People’s Hospital of Nanjing Medical University, Taizhou School of Clinical Medicine, Nanjing Medical University, Taizhou, Jiangsu, China; 5 School of Public Health, National Vaccine Innovation Platform, Nanjing Medical University, Nanjing, China

**Keywords:** COVID-19, inactivated COVID-19 vaccine, multi-omics, proteomics, pulmonary tuberculosis, systems vaccinology, transcriptomics

## Abstract

**Introduction:**

Patients with pulmonary tuberculosis (PTB) may experience persistent immune dysregulation during anti-tuberculosis treatment, yet vaccine evaluation in this population has largely relied on humoral endpoints. Whether preserved antibody responses reflect coordinated systems-level immune activation after inactivated COVID-19 vaccination remains unclear. This study used systems vaccinology to identify multi-omic immune signatures induced by vaccination in PTB patients.

**Methods:**

We compared PTB patients who had completed at least 3 months of anti-tuberculosis therapy with healthy controls. This nested subcohort analysis was derived from a phase IV multicenter randomized controlled trial that evaluated vaccine safety and immunogenicity in PTB patients and healthy participants. Paired plasma samples for proteomic profiling using the Olink Explore 3072 platform and peripheral blood mononuclear cells (PBMCs) for transcriptomic sequencing were collected at baseline (Day 0, pre-vaccination) and Day 56 (28 days post-second dose). Proteomic analysis included 38 PTB patients and 38 healthy controls, while transcriptomic profiling was performed on a subset of 20 participants per group. We compared neutralizing antibody responses, differential molecular signatures, inferred immune-cell fractions, and antibody-protein correlations between the two cohorts. Within each group, we further characterized longitudinal molecular alterations from Day 0 to Day 56.

**Results:**

After two doses of inactivated COVID-19 vaccine, PTB patients and healthy controls achieved comparable neutralizing antibody titers and similar seroconversion rates. However, the two cohorts exhibited markedly distinct baseline immune landscapes. Prior to vaccination, PTB patients had widespread depletion of plasma proteins enriched in vesicle transport and phagocytosis pathways, accompanied by pro-inflammatory transcriptional reprogramming. After vaccination, healthy controls showed broad proteomic and transcriptomic remodeling, whereas PTB patients displayed minimal molecular perturbation and limited inferred immune-cell changes. Exploratory covariate-adjusted partial Spearman protein-antibody correlation analyses suggested a predominance of nominal negative associations in PTB patients, involving proteins related to inflammatory regulation, intracellular trafficking, and phagocytosis; no individual association survived false-discovery-rate correction.

**Conclusion:**

Despite comparable short-term neutralizing antibody responses, PTB patients receiving anti-tuberculosis treatment exhibited attenuated and distinct systems-level proteomic and transcriptomic remodeling after inactivated COVID-19 vaccination compared with healthy controls.

**Clinical Trial Registration:**

https://clinicaltrials.gov/study/NCT05148949, identifier NCT05148949.

## 1 Introduction

The World Health Organization Global Vaccine Action Plan has emphasized that the benefits of immunization should be extended to all vulnerable populations, including individuals with chronic diseases or immune dysfunction (1). However, conventional vaccine clinical trials predominantly recruit healthy volunteers, whereas patients with underlying disorders and immunocompromised individuals, such as those undergoing anti-tuberculosis treatment, are frequently excluded from trial cohorts.

Pulmonary tuberculosis (PTB) is a chronic infectious disease characterized by persistent immune activation, altered innate immune function, and impaired adaptive immune regulation (2). Such immune disturbances are hypothesized to modify vaccine-induced immune responses, including the magnitude and coordination of vaccine-triggered molecular and cellular cascades (3). Existing evidence suggests that PTB may be associated with altered humoral and cellular immune profiles, which could modify vaccine-induced immune responses (4, 5).

However, relying solely on a single antibody titer or cellular marker often fails to fully characterize heterogeneous vaccine-elicited immune responses in small-cohort studies (6). This challenge is further illustrated by COVID-19 vaccines, for which validated immune surrogate endpoints for clinical protection remain poorly defined. Systems immunology integrates high-throughput omics technologies with computational modeling to construct an integrative framework for unraveling vaccine-initiated immune networks and signaling cascades (7). As a derivative discipline, systems vaccinology leverages multi-omics datasets spanning transcriptomics, proteomics, metabolomics and single-cell phenotyping to pinpoint predictive molecular signatures of vaccine potency and elucidate the mechanistic basis underlying long-lasting protective immunity (8). Unlike single immune biomarkers, these multi-layered omics tools can capture early transcriptional and protein network signals predictive of subsequent immune outcomes, providing comprehensive, dynamic insights to guide vaccine development and evaluation.

We previously completed a randomized controlled trial comparing two-dose inactivated COVID-19 vaccination between PTB patients on anti-tuberculosis therapy and healthy controls (9). After two doses of inactivated COVID-19 vaccine, comparable neutralizing antibody titers and seroconversion rates were observed across the two groups. Despite equivalent humoral immunity, the underlying molecular immune landscape after vaccination in PTB patients remains uncharacterized. To interpret the systems-level molecular correlates of these earlier clinical observations, this study employed a systems vaccinology strategy to analyze longitudinal plasma proteome and peripheral blood transcriptome profiles from participants enrolled in the aforementioned trial.

## 2 Materials and methods

### 2.1 Cohort design and study participants

This nested subcohort analysis originated from a previously completed phase IV multicenter randomized controlled trial evaluating the safety and immunogenicity of an inactivated COVID-19 vaccine among patients with PTB (9). Briefly, the parent trial enrolled adults with PTB who were receiving anti-tuberculosis treatment after completing at least 3 months of intensive-phase therapy, as well as healthy controls aged 18–75 years in Jiangsu Province, China, between December 22, 2021, and March 6, 2023.

For proteomic profiling, 38 of the 40 enrolled healthy controls had available paired Day 0 and Day 56 plasma samples and were included in the Olink analysis. An equal number of PTB patients were selected from the parent trial among participants with paired plasma samples, complete neutralizing antibody measurements, and proteomic quality-control eligibility. Propensity-score matching was performed using sex and age. BMI was not included in the initial matching procedure because of the limited size of the healthy-control sampling pool and the need to preserve paired omics sample size; however, BMI, age, and sex were included as covariates in subsequent between-group omics comparisons and protein-antibody correlation analyses. Covariate balance before and after matching is summarized in **Supplementary Table S2**. For transcriptomic profiling, 20 PTB patients and 20 healthy controls were selected from the proteomic cohort for paired PBMC RNA-sequencing analysis, as summarized in **Supplementary Table S1**. Subcohort selection occurred after neutralizing-antibody results were available, but antibody magnitude was not used as a selection criterion. For transcriptomic profiling, 20 participants per group were randomly selected from the proteomic cohort independently of antibody magnitude.

In the parent trial, PTB participants were scheduled for three vaccine doses administered on Days 0, 28 and 56, whereas healthy controls received a two-dose primary series on Days 0 and 28. All transcriptomic and proteomic profiling in this work relied on paired biospecimens collected during the two-dose primary vaccination period (**Figure 1**).

**Figure 1:**
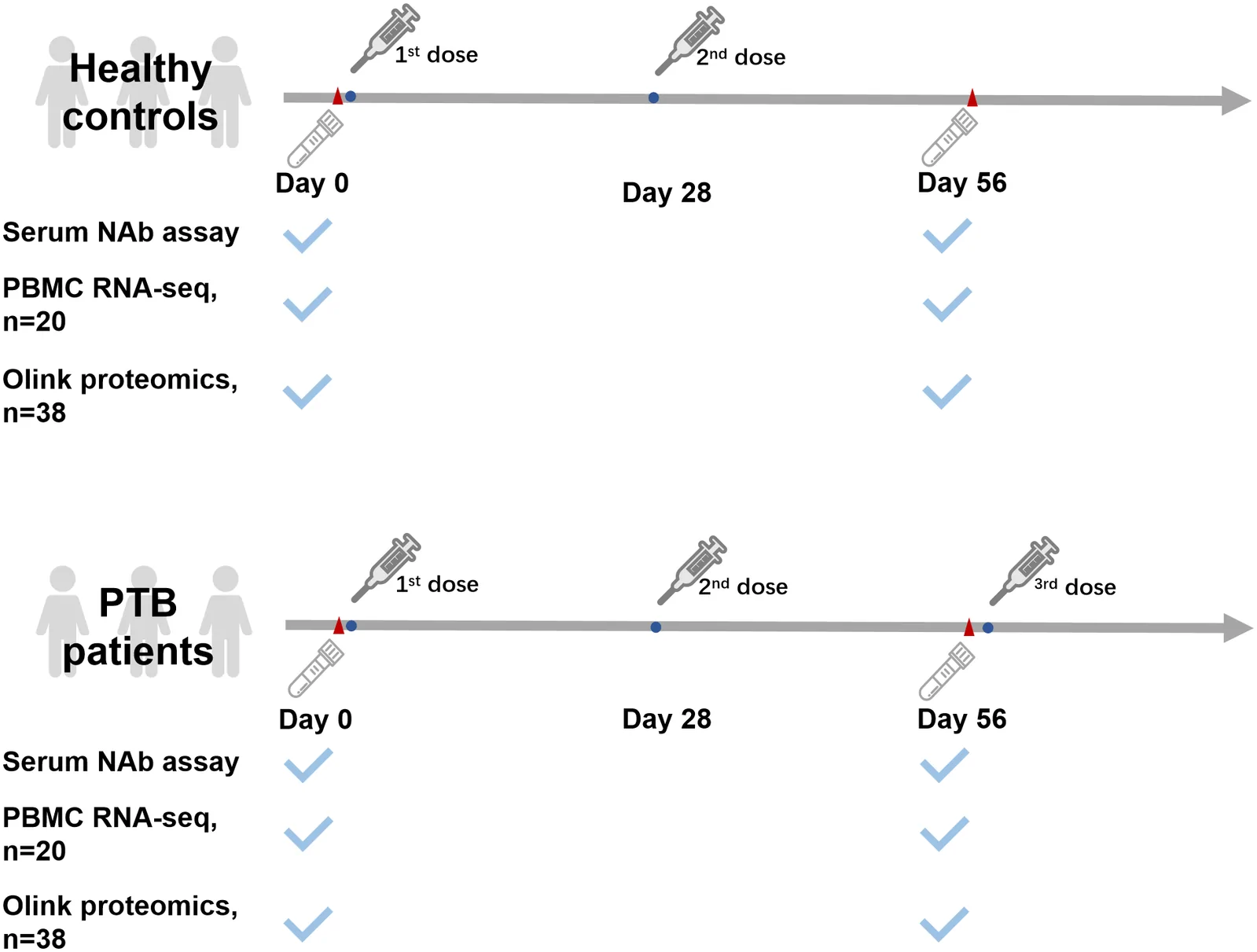
Vaccination schedule and paired biospecimen collection for multi-omics analyses. Healthy controls received two doses of inactivated COVID-19 vaccine on Days 0 and 28, whereas patients with pulmonary tuberculosis (PTB) receiving anti-tuberculosis treatment were scheduled to receive three doses on Days 0, 28, and 56 in the parent trial. Paired biospecimens were collected at baseline (Day 0, before vaccination) and Day 56. For PTB patients, Day 56 biospecimens were collected before administration of the protocol-specified third dose and therefore reflected immune responses 28 days after the second dose. Serum samples were used for neutralizing antibody assessment, PBMC samples were used for RNA-sequencing transcriptomic profiling, and plasma samples were used for Olink proteomic profiling. PBMC RNA-seq was performed in 20 participants per group, and Olink proteomic profiling was performed in 38 participants per group. PTB, pulmonary tuberculosis; PBMC, peripheral blood mononuclear cell.

All transcriptomic samples had paired PBMC specimens collected at Day 0 and Day 56 and met RNA-sequencing quality-control requirements. In the omics cohort, Day 0 samples were collected from December 22, 2021, to August 10, 2022, and Day 56 samples were collected from February 17 to October 6, 2022. China maintained the dynamic zero-COVID strategy throughout this sampling window, and no participant included in the omics analyses had documented SARS-CoV-2 infection between Day 0 and Day 56. For PTB participants, Day 56 blood samples were collected before the third vaccine dose administered on the same day. Samples collected after the third dose were not analyzed because post-third-dose follow-up overlapped the widespread Omicron infection wave that followed relaxation of national control measures in December 2022, making vaccination and natural-infection effects difficult to disentangle. Therefore, the Day 56 omics and antibody measurements represented responses 28 days after the second dose and were not affected by the third dose.

This study was approved by the Institutional Review Board of the Jiangsu Provincial Center for Disease Control and Prevention. Written informed consent was obtained from all participants prior to enrollment. This trial was conducted following the principles of the Declaration of Helsinki and Good Clinical Practice Guidelines.

### 2.2 Measurement of antigen-specific serum antibodies

Neutralizing antibody data were derived from the parent clinical trial and were used in the present nested omics study as the humoral immunogenicity reference endpoint (9). Live virus neutralizing antibodies were determined using a cytopathic effect (CPE)-based microneutralization assay using wild-type SARS-CoV-2 virus, as previously described (10). This endpoint was retained because it was prespecified in the parent trial and was homologous to the ancestral-strain whole-virion antigen in CoronaVac; it was not intended to estimate protection against Omicron-lineage variants. For samples with no detectable neutralizing activity at the lowest tested dilution (1:4), the titer was assigned as half the limit of quantification (1:2) for statistical analysis. Seroconversion was defined as antibody titers from seronegative (<1:4) at pre-vaccination baseline to ≥1:16 at Day 56, or a minimum four-fold increase in neutralizing antibody titers from Day 0 to Day 56 after vaccination.

### 2.3 RNA-sequencing

Total RNA was isolated from PBMCs using the RNeasy Mini Kit (Qiagen, Germany) according to the manufacturer’s instructions. RNA integrity was evaluated with an Agilent 2100 Bioanalyzer. Samples with an RNA integrity number (RIN) ≥ 7.0 were eligible for library construction. Strand-specific RNA libraries were prepared using the Illumina TruSeq Stranded mRNA Library Prep Kit and sequenced on the Illumina NovaSeq 6000 platform to generate 150 bp paired-end reads. Raw sequencing reads were trimmed and quality-filtered using fastp. Clean reads were aligned to the human reference genome (GRCh38) via the STAR aligner. Gene-level expression counts were quantified using featureCounts, and the resulting count matrix was used for subsequent differential expression analysis.

### 2.4 Proteomic data measurements

Plasma proteomic profiling was performed using the Olink Explore 3072 platform in accordance with the manufacturer’s standard instructions. Raw proteomic data underwent quality control and normalization according to Olink-recommended procedures. Proteins that failed quality control or were detected in fewer than half of the study samples were excluded, leaving 2,902 unique proteins with valid NPX values for downstream analyses. According to laboratory records, samples were randomly allocated across three assay plates; both study groups and both visits were represented on every plate (**Supplementary Table S4**). Plate was not included as a covariate in the primary omics models.

### 2.5 Data analysis

All statistical analyses were performed using R software (version 4.4.3). For each protein, baseline between-group differences in NPX values were assessed using a multivariable linear model with study group as the primary variable and age, sex, and BMI as covariates. Baseline transcriptomic comparisons were performed using DESeq2 with a design matrix including study group, age, sex, and BMI. Within-group longitudinal proteomic changes from Day 0 to Day 56 were assessed using paired Wilcoxon signed-rank tests followed by Benjamini-Hochberg FDR correction. Within-group longitudinal transcriptomic changes were assessed separately in each study group using DESeq2 models that included participant as a blocking factor and visit (Day 56 vs. Day 0) as the contrast of interest, thereby accounting for the paired design. Differentially abundant proteins were defined as proteins with FDR-adjusted P<0.05. Differentially expressed genes were identified using DESeq2 and defined as genes with FDR-adjusted P<0.05 and |log2 fold change|>1. Different thresholds were used because Olink NPX values are normalized log2-scale protein abundance measures with a narrower dynamic range, whereas RNA-seq count data have broader fold-change dispersion; therefore, an additional |log2 fold change| > 1 threshold was applied to transcriptomic analyses.

Principal component analysis (PCA) was performed using the FactoMineR package after centering and scaling variables. PCA analyses were conducted separately for each omics platform to visualize baseline between-group distributions and longitudinal Day 0-to-Day 56 changes within each study group.

For each ranked gene or protein list, GSEA was performed using 1,000 permutations to identify coordinated pathway-level alterations. Pathways were considered significant at FDR-adjusted P < 0.05, and the direction of enrichment was interpreted according to the normalized enrichment score.

CIBERSORT with the LM22 signature matrix and 1,000 permutations was applied to bulk PBMC RNA-seq data to computationally estimate relative immune-cell fractions. All samples met the prespecified CIBERSORT deconvolution quality-control criterion (P<0.05) and were retained for descriptive analyses; no sample was excluded for poor deconvolution performance. CIBERSORT P value, correlation, and RMSE are reported in **Supplementary Table S5**; cell types detected as nonzero in fewer than half of samples were not carried forward to comparative visualization.

Associations between Day 56 plasma NPX values and log2-transformed Day 56 neutralizing antibody titers were first assessed separately within PTB patients and healthy controls using partial Spearman correlation adjusted for age, sex, and BMI. Benjamini-Hochberg FDR correction was applied across all 2,902 tested proteins within each group. **Figure 2** visualizes nominal P<0.05 patterns for exploratory purposes; associations that did not survive FDR correction, together with downstream enrichment and interaction-network analyses based on those nominal sets, were interpreted as exploratory and hypothesis-generating. Complete adjusted results are provided in **Supplementary Table S6**.

**Figure 2:**
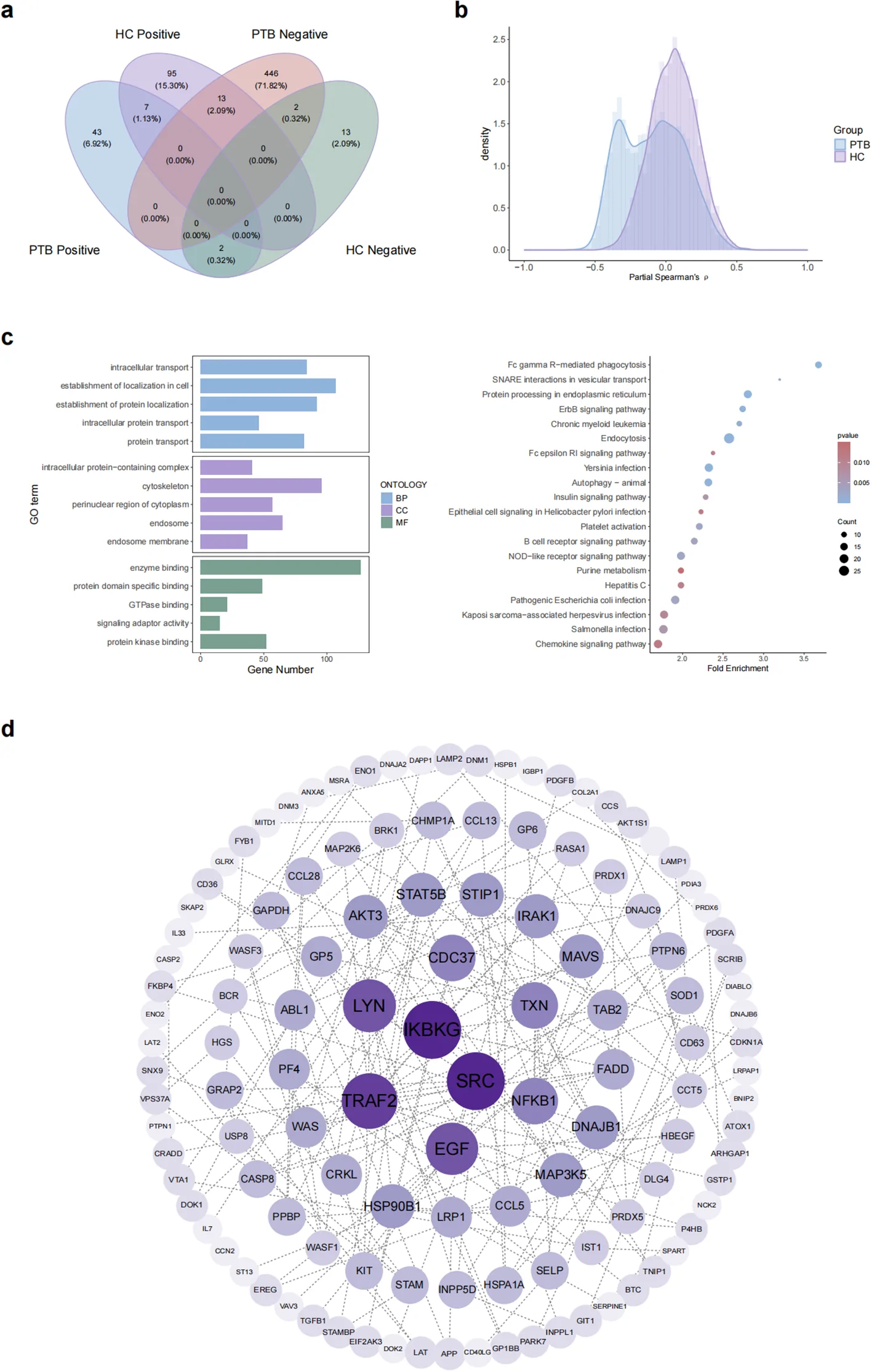
Exploratory antibody–protein association patterns in PTB patients and healthy controls. **(a)** Numbers of plasma proteins showing exploratory nominal positive or negative partial Spearman associations with Day 56 neutralizing antibody titers in PTB patients and healthy controls after adjustment for age, sex, and BMI. Percentages shown in the four-set Venn diagram use the 621 proteins in the union of the four nominal direction-specific sets as the denominator. **(b)** Density distribution of adjusted partial Spearman correlation coefficients between Day 56 plasma protein abundance and log2-transformed neutralizing antibody titers. **(c)** Functional enrichment of proteins nominally negatively associated with neutralizing antibody titers in PTB patients. **(d)** Protein interaction network of PTB-specific proteins nominally negatively associated with neutralizing antibody titers. Benjamini-Hochberg FDR correction was applied across 2,902 tested proteins within each group. No individual association survived FDR<0.05; nominal associations and downstream pathway/network analyses were interpreted as exploratory and hypothesis-generating. Proteomic cohort: PTB n=38, HC n=38.

## 3 Results

### 3.1 Cohort characteristics and antibody responses

The plasma proteomic cohort comprised 38 PTB patients and 38 healthy controls, and the PBMC transcriptomic subset included 20 participants per group. Specifically, PTB patients were younger (mean age 42.97 vs. 52.34 years, P = 0.013) and had lower BMI compared with healthy controls. Sex and comorbidities were balanced (**Table 1**). Additional PTB-specific clinical and treatment characteristics, including cavitary disease, microbiological positivity, rifampicin resistance, treatment regimen, treatment duration, and treatment outcome, are summarized in **Supplementary Table S3**. Treatment duration and regimen varied among PTB participants, indicating that treatment exposure was not completely uniform across the PTB group.

**Table 1:** Baseline characteristics of PTB and healthy control analysis cohorts.

Characteristic	Level	PTB patients	Healthy controls	P
Proteomic analysis cohort				
n		38	38	
Age, mean (SD)		42.97 (18.10)	52.34 (11.23)	0.013
Sex, n (%)	Female	17 (44.7)	19 (50.0)	0.818
	Male	21 (55.3)	19 (50.0)	
BMI, n (%)	<18.5	5 (13.2)	0 (0.0)	0.011
	18.5–23.9	23 (60.5)	18 (47.4)	
	≥24	10 (26.3)	20 (52.6)	
Comorbidity, n (%)	No	27 (71.1)	28 (73.7)	1.000
	Yes	11 (28.9)	10 (26.3)	
Transcriptomic analysis cohort				
n		20	20	
Age, mean (SD)		50.05 (18.76)	56.25 (10.70)	0.490
Sex, n (%)	Female	10 (50.0)	10 (50.0)	1.000
	Male	10 (50.0)	10 (50.0)	
BMI, n (%)	<18.5	3 (15.0)	0 (0.0)	0.051
	18.5–23.9	13 (65.0)	10 (50.0)	
	≥24	4 (20.0)	10 (50.0)	
Comorbidity, n (%)	No	15 (75.0)	14 (70.0)	1.000
	Yes	5 (25.0)	6 (30.0)	

Data are presented as mean (SD) or n (%). For continuous variables, normality was assessed using the Shapiro-Wilk test. Normally distributed variables were compared using Student’s t-test, whereas non-normally distributed variables were compared using the Mann-Whitney U test. Categorical variables were compared using the χ2 test or Fisher’s exact test when expected cell counts were small. The test used for each **Table 1** variable is provided in **Supplementary Table S7**. BMI was categorized according to Chinese adult criteria: underweight, <18.5 kg/m2; normal weight, 18.5-23.9 kg/m2; overweight/obesity, >=24 kg/m2. PTB, pulmonary tuberculosis; HC, healthy controls; BMI, body mass index.

Using neutralizing antibody data from the previously published parent trial (9), both PTB patients and healthy controls showed increased SARS-CoV-2-specific neutralizing antibody titers from pre-vaccination baseline to Day 56. At Day 56, the GMT of neutralizing antibodies was 36.8 (95% CI: 30.3-44.7) in PTB patients and 31.4 (95% CI: 22.8-43.2) in healthy controls (P = 0.462). Seroconversion rates were also comparable with 83.9% (95% CI: 77.5-89.1) in PTB patients versus 87.5% (95% CI: 73.2-95.8) in healthy controls (P = 0.697). These results indicate that PTB patients on anti-tuberculosis treatment achieve humoral vaccine responses comparable to healthy individuals following two-dose inactivated COVID-19 vaccination.

### 3.2 Baseline immune profile comparison

Principal component analysis (PCA) revealed largely overlapping global gene and protein expression patterns at the pre-vaccination baseline on Day 0 (**Figures 3a, b**), indicating global similarity between the two cohorts. However, differential analysis identified 450 proteins (445 downregulated) and 858 genes (359 downregulated) in PTB patients at Day 0 (**Figures 3c, d**). Differential proteins were enriched in vesicle-mediated transport and phagocytosis pathways (**Supplementary Figure S1a**), whereas differentially expressed genes were enriched in chemotaxis/taxis, cytokine-related inflammatory programs, acute inflammatory responses, and innate immune activation (**Supplementary Figure S1b**). In addition, GSEA revealed negative enrichment of complement and coagulation cascades, neutrophil extracellular trap formation, oxidative phosphorylation, and phagosome pathways in PTB patients (**Supplementary Figure S1c**).

**Figure 3:**
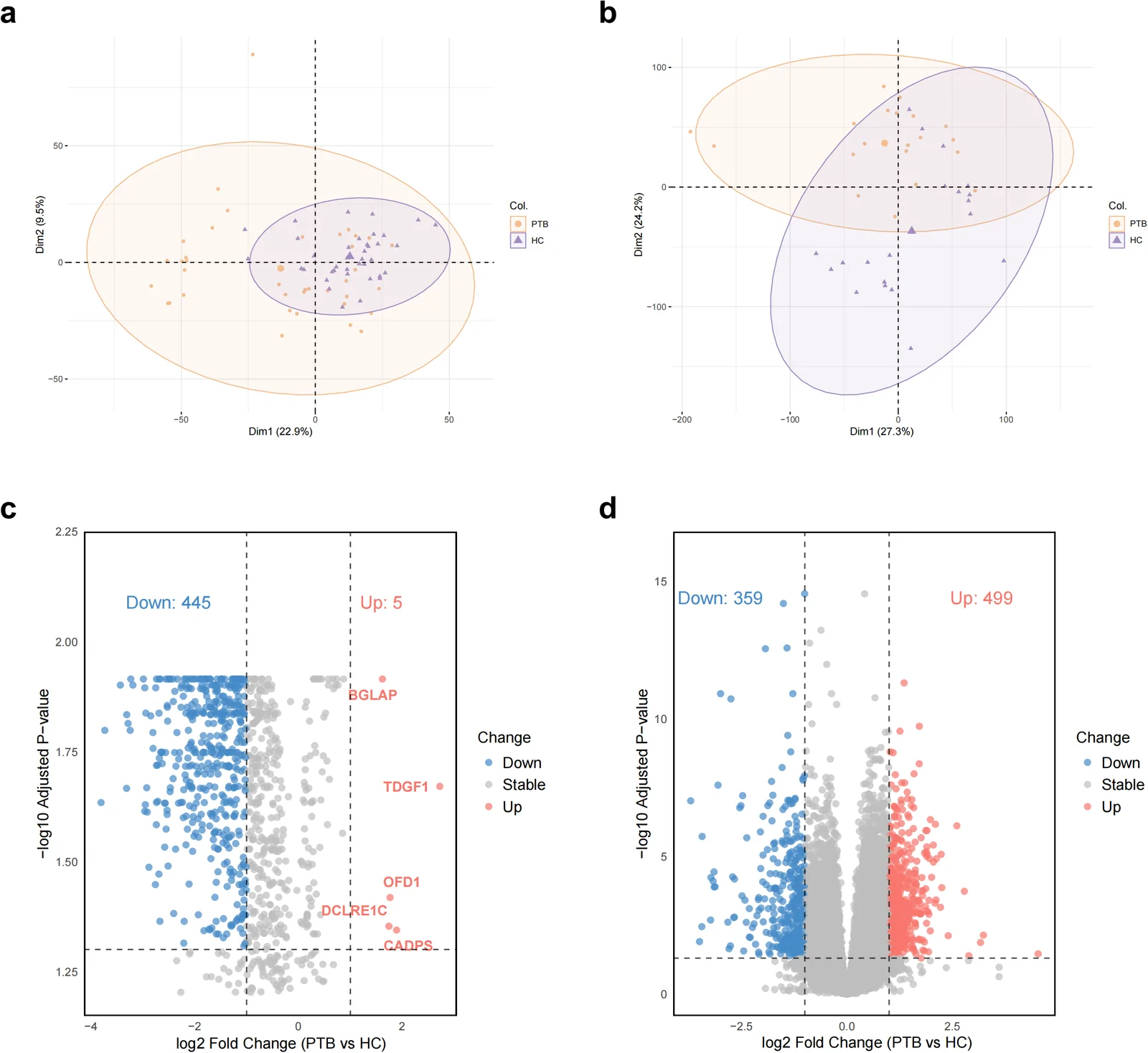
Baseline multi-omics immune features in PTB patients and healthy controls. **(a)** Principal component analysis (PCA) of plasma proteomic profiles at Day 0. **(b)** PCA of PBMC transcriptomic profiles at Day 0. **(c)** Volcano plot showing baseline differentially abundant plasma proteins between PTB patients and healthy controls. **(d)** Volcano plot showing baseline differentially expressed genes between PTB patients and healthy controls. For volcano plots, log2 fold change was calculated as PTB versus healthy controls. Differentially abundant proteins were defined as FDR-adjusted P<0.05. Differentially expressed genes were defined as FDR-adjusted P<0.05 and |log2 fold change|>1. Proteomic cohort: PTB n=38, HC n=38; transcriptomic cohort: PTB n=20, HC n=20.

In terms of immune cell deconvolution, PTB patients had significantly higher proportions of naive B cells (5.12 ± 2.42% vs. 2.25 ± 1.60%, P_FDR_ < 0.001) and resting CD4^+^ memory T cells (13.20 ± 8.71% vs. 4.93 ± 6.31%, P_FDR_ = 0.001), while healthy controls had elevated proportions of monocytes (33.56 ± 9.62% vs. 21.93 ± 10.43%, P_FDR_ = 0.016) and activated CD4^+^ memory T cells (1.98 ± 2.55% vs. 0.52 ± 0.80%, P_FDR_ = 0.001) (**Supplementary Figure S2**). Together, these pre-vaccination proteomic, transcriptomic, and inferred immune-cell data suggest pathway-specific immune alterations in PTB patients.

### 3.3 Proteomic response to vaccination

In healthy individuals, PCA showed a clear temporal separation between baseline and Day 56 proteomic profiles along the first principal component (Dim1, 24.4% variance), consistent with broad Day 0-to-Day 56 changes in the measured plasma proteomic profiles following two-dose vaccination (**Figure 4a**). Conversely, PTB patients exhibited nearly complete overlap between pre- and post-vaccination proteomic profiles, consistent with smaller Day 0-to-Day 56 changes within the measured Olink protein panel (**Figure 4b**).

**Figure 4:**
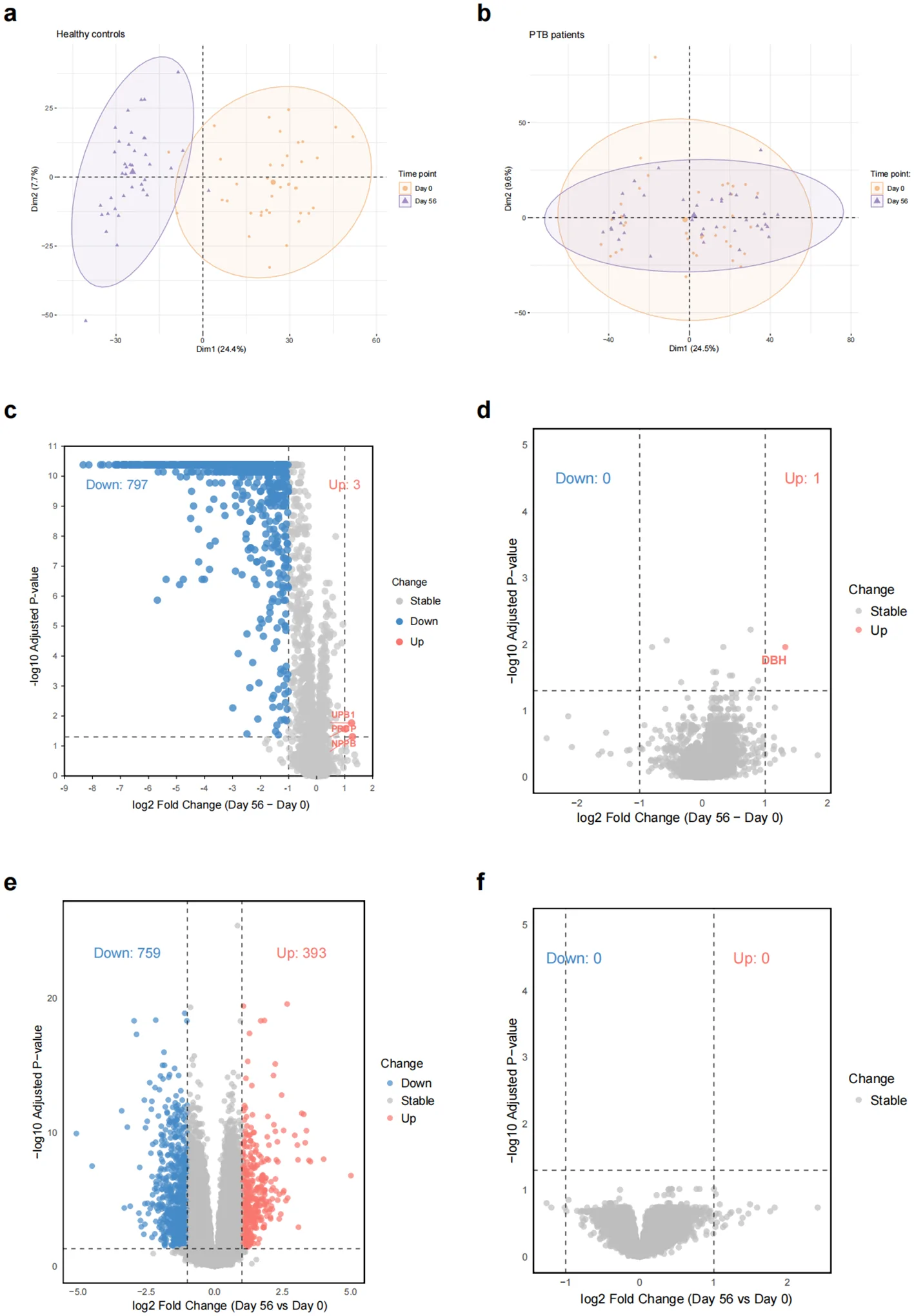
Divergent proteomic and transcriptomic remodeling after vaccination in healthy controls and PTB patients. **(a, b)** Principal component analysis of plasma proteomic profiles from Day 0 to Day 56 in healthy controls and PTB patients. **(c, d)** Volcano plots showing longitudinal plasma proteomic changes from Day 0 to Day 56 in healthy controls and PTB patients. Protein changes were calculated as ΔNPX values, defined as NPX at Day 56 minus NPX at Day 0. **(e, f)** Volcano plots showing longitudinal transcriptomic changes from Day 0 to Day 56 in healthy controls and PTB patients. Increased and decreased proteins were identified using paired comparisons followed by FDR correction. Longitudinal transcriptomic changes were identified using paired DESeq2 models including participant as a blocking factor and visit as the contrast of interest. Differentially expressed genes were defined as FDR-adjusted P < 0.05 and |log2 fold change| > 1. Proteomic cohort: PTB n = 38, HC n = 38. Transcriptomic cohort: PTB n = 20, HC n = 20.

Consistent with the PCA results, paired longitudinal analysis confirmed extensive plasma proteomic remodeling in healthy controls after vaccination, with 797 proteins decreased and 3 increased at Day 56 relative to Day 0 (**Figure 4c**). In comparison, PTB patients exhibited minimal change, with only marginal upregulation of dopamine β-hydroxylase (**Figure 4d**). Collectively, these findings indicate that, despite comparable neutralizing antibody responses at Day 56, PTB patients showed markedly attenuated systemic proteomic remodeling after inactivated COVID-19 vaccination.

### 3.4 Transcriptomic response to vaccination

From Day 0 to Day 56, healthy controls underwent extensive transcriptomic reprogramming following two-dose vaccination, along with 1,152 differential genes (393 upregulated, 759 downregulated; **Figure 4e**). The downregulated genes were enriched in pathways controlling cellular metabolism and biosynthetic pathways, consistent with canonical metabolic reprogramming during immune cell activation and adaptive differentiation (**Supplementary Figure S3a**). Simultaneously, upregulated genes were enriched in IL-17 signaling, TNF signaling, and TGF-β-related signaling (**Supplementary Figure S3b**).

In contrast, PTB patients showed a narrower log2 fold-change distribution and fewer genes meeting the prespecified differential-expression threshold (**Figure 4f**; **Supplementary Figures S4a, b**). These transcriptomic findings suggest that PTB patients showed limited systemic transcriptional remodeling after vaccination, despite mounting serologically detectable antibody responses comparable to healthy controls.

### 3.5 Changes in immune cell composition

In healthy controls, the computationally estimated fraction of resting CD4^+^ memory T cells increased from 4.93 ± 6.31% at Day 0 to 13.91 ± 5.36% at Day 56 (PFDR < 0.001; **Figure 5a**; **Supplementary Table S5**), consistent with transcriptomic enrichment of T-cell receptor signaling. Most other inferred lymphocyte populations remained largely unchanged, suggesting that vaccination was associated with selective remodeling of specific T cell subsets rather than global immune cell perturbation (**Supplementary Figure S5**).

**Figure 5:**
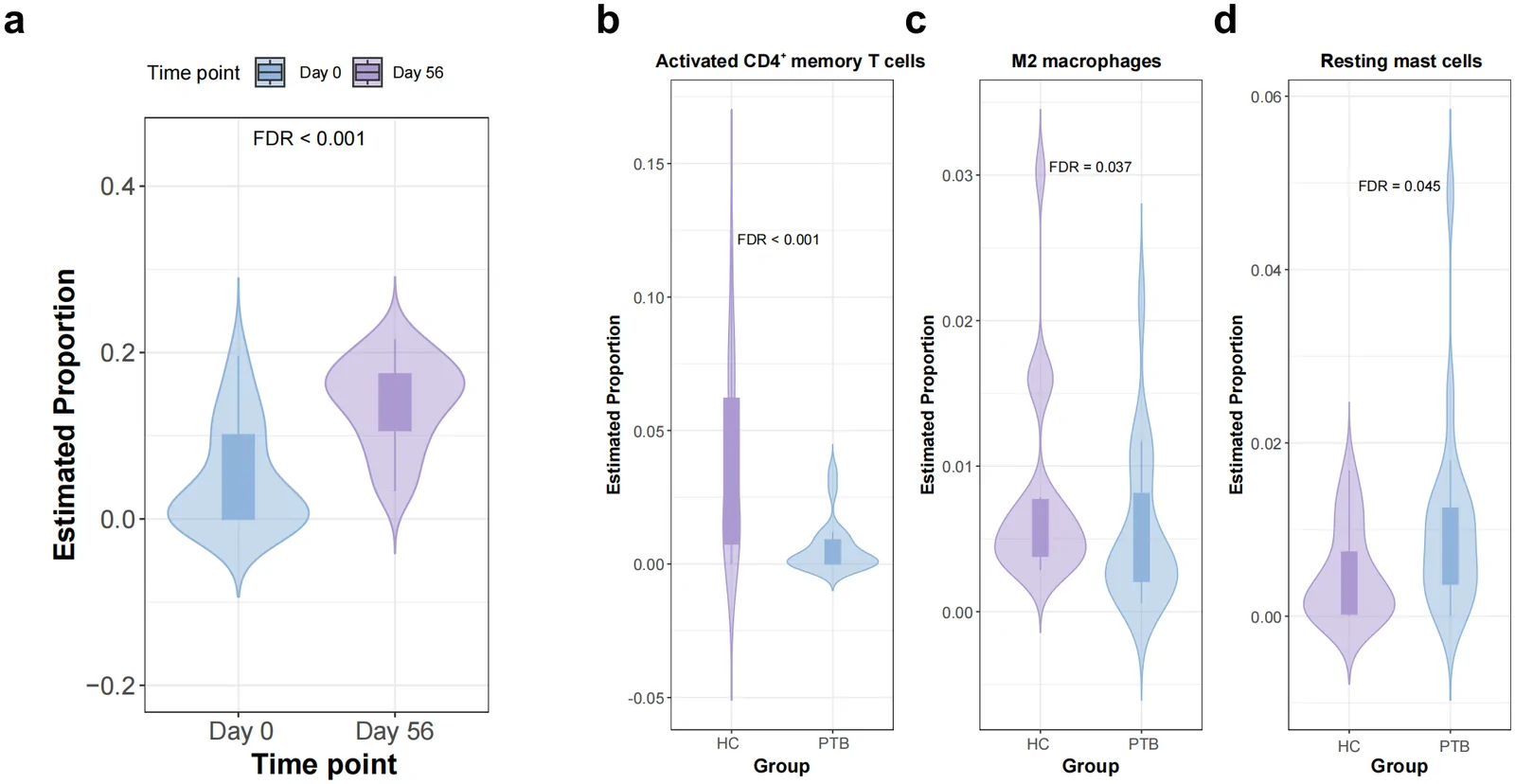
Immune-cell compositional remodeling after vaccination differs between healthy controls and PTB patients. Immune-cell fractions were computationally estimated from PBMC transcriptomic data using CIBERSORT with the LM22 signature matrix. **(a)** Change in inferred resting CD4+ memory T-cell fractions from Day 0 to Day 56 within healthy controls. **(b–d)** Differential inferred immune-cell fractions between PTB patients and healthy controls at Day 56, including activated CD4+ memory T cells, M2 macrophages, and resting mast cells. P values were corrected for multiple comparisons using the FDR method. Transcriptomic cohort: PTB n=20, HC n=20.

In contrast, PTB patients exhibited no significant changes in immune-cell subsets (**Supplementary Figure S6**), consistent with the blunted molecular responses described above. Direct cross-sectional comparison at Day 56 revealed distinct post-vaccination immune-cell landscapes between the two groups, with healthy controls showing higher proportions of activated CD4^+^ memory T cells and M2-polarized macrophages, while PTB patients showed increased resting mast cells (all P_FDR_ < 0.05; **Figures 5b–d**). As shown in **Supplementary Figure S7**, these key cell subsets exhibited well-segregated distributions between cohorts, tightly aligning with their molecular phenotypes.

### 3.6 Differential antibody–protein correlations

To further characterize the Day 56 humoral response, we examined covariate-adjusted partial Spearman correlations between plasma protein abundance and neutralizing antibody titers separately in PTB patients and healthy controls. At the nominal P<0.05 threshold, 462 proteins showed negative associations in PTB patients; 446 occupied the PTB-negative-only region of the four-set Venn diagram, corresponding to 446 of the 621 proteins in the union of the four nominal direction-specific sets (71.82%) (**Figure 2a**). Healthy controls showed fewer nominal negative associations and a more balanced correlation pattern. No individual protein-antibody association survived Benjamini-Hochberg correction at FDR<0.05 (**Supplementary Table S6**); therefore, all displayed nominal patterns were interpreted as exploratory and hypothesis-generating.

Notably, correlation coefficient distributions were left-shifted in PTB patients, while centered around zero in healthy controls (**Figure 2b**). Minimal overlap existed between the two groups’ nominal associations; virtually no protein was significantly correlated with antibody levels in both groups. Among proteins meeting the nominal P<0.05 threshold in the PTB group, exploratory enrichment analysis highlighted cytosolic transport, endoplasmic reticulum protein processing, Fc receptor signaling, and phagocytosis-related pathways (**Figure 2c**). Exploratory protein–protein interaction analysis of these nominally associated proteins identified a subnetwork containing several NF-κB-related nodes, including IKBKG/NEMO, NFKB1, TRAF2, SRC, EGF, and LYN (**Figure 2d**).

## 4 Discussion

This multi-omics study identified differences between conventional serological endpoints and systems-level immune programming in individuals with PTB following inactivated COVID-19 vaccination (11). Although PTB patients achieved neutralizing antibody titers and seroconversion rates statistically comparable to healthy controls at Day 56 (9), they showed smaller longitudinal changes in the measured proteomic and bulk-PBMC transcriptomic datasets, together with fewer changes in CIBERSORT-estimated immune-cell fractions. Thus, the measured Day 56 neutralizing-antibody endpoint did not fully reflect the between-group differences observed in these systems-level assays. The functional and clinical significance of these differences remains unknown (12).

This divergence is important in the context of systems vaccinology. Previous systems-level studies have shown that effective vaccine responses are typically accompanied by coordinated activation of innate, transcriptional, cellular, and humoral programs, rather than by antibody production alone (13–15). In healthy controls in our study, vaccination induced broad proteomic shifts and substantial transcriptomic changes, including downregulation of metabolic and biosynthetic programs and upregulation of inflammatory and immune signaling pathways (16, 17), reflecting metabolic remodeling, T-cell receptor signaling, and innate-adaptive crosstalk during vaccine-induced immune activation (13). By contrast, PTB patients showed nearly complete overlap between pre- and post-vaccination proteomic profiles and minimal transcriptomic perturbation. These observations are compatible with an association between PTB/treatment status and attenuated systems-level molecular changes after vaccination, but the underlying mechanisms and functional significance remain to be established (18).

Baseline immune differences may provide a biological context for the attenuated systems-level remodeling observed in PTB patients, but they should not be interpreted as definitive evidence of a causal mechanism. At Day 0, PTB patients displayed marked downregulation of plasma proteins involved in vesicle organization, intracellular and endosomal transport, and phagocytosis-related pathways. These pathways are closely connected to antigen uptake, intracellular trafficking, antigen processing, and communication between innate and adaptive immune compartments (19). Baseline transcriptomic differences further revealed a state of chemotaxis, cytokine-related inflammation, and acute inflammatory programs (20). Together, these baseline alterations suggest an altered pre-vaccination immune landscape that may be associated with reduced downstream molecular responsiveness to vaccination; however, this hypothesis requires validation in mechanistic and antigen-specific cellular studies (11).

The partial discordance between plasma proteomic and PBMC transcriptomic changes should also be interpreted biologically rather than as a contradiction. The plasma proteome reflects systemic circulating proteins shaped by secretion, cellular turnover, tissue release, and systemic inflammation (21), whereas the PBMC transcriptome captures gene-expression programs within a restricted immune cell compartment (22). Therefore, the combination of reduced circulating proteins involved in trafficking and phagocytosis and inflammatory transcriptional activation may reflect different layers of PTB-associated immune remodeling. Such compartmentalized differences reinforce the value of integrated multi-omics approaches in this setting, because single-layer immune measurements could miss such compartment-specific alterations (7).

The immune-cell deconvolution results provide computational support for differences in post-vaccination immune-cell composition, but these estimates should be interpreted cautiously because they were inferred from bulk PBMC transcriptomic data rather than directly measured by flow cytometry or single-cell profiling. Healthy controls exhibited an increase in inferred resting CD4^+^ memory T-cell fractions after vaccination and higher inferred activated CD4^+^ memory T-cell and M2 macrophage fractions at Day 56, consistent with computational evidence of adaptive immune remodeling and coordinated T-cell help (23). In contrast, PTB patients showed no significant longitudinal immune-cell changes and higher resting mast-cell fractions at Day 56, consistent with an altered innate/regulatory profile rather than definitive evidence of productive cellular immune remodeling (24). These cellular patterns are consistent with the proteomic and transcriptomic data, collectively suggesting that healthy controls developed a more coordinated adaptive immune remodeling pattern, whereas PTB patients remained relatively transcriptionally and cellularly quiescent after vaccination.

Exploratory analysis of proteins meeting the nominal P<0.05 threshold suggested different antibody-protein association patterns between the two groups. In the PTB group, nominally negatively associated proteins were enriched in intracellular transport, endoplasmic reticulum protein processing, Fc receptor-related signaling, and phagocytosis-related processes. Exploratory protein–protein interaction analysis identified a network containing several NF-κB-related nodes (25–27). However, no individual protein-antibody association survived FDR correction. Therefore, these enrichment and network patterns should be interpreted solely as hypothesis-generating observations requiring validation in larger independent cohorts.

Several limitations should be acknowledged. First, only baseline and Day 56 time points were included, so early innate events after the first and second vaccine doses were not captured. This is relevant because early transcriptional and innate immune signatures have been shown to predict later vaccine-induced antibody responses in systems vaccinology studies (11, 15). Second, immune cell fractions were computationally inferred rather than measured by flow cytometry or single-cell profiling (28). No orthogonal flow-cytometric validation, antigen-induced activation-marker assay, intracellular cytokine staining, ELISpot, tetramer staining, or other vaccine antigen-specific cellular immune assay was available; therefore, the cellular findings should be interpreted as computational and systems-level observations rather than direct evidence of altered vaccine-specific T-cell magnitude, phenotype, or function. Third, this study did not directly assess long-term antibody durability (29), Fc-mediated effector functions (12), antigen-specific T-cell responses, or clinical protection (23). Because all PTB participants were receiving anti-tuberculosis therapy, the observed proteomic and transcriptomic differences may reflect both TB-related immune alterations and treatment-related effects of anti-tuberculosis drugs; this potential confounding could not be fully separated in the present study. In addition, the neutralization assay measured ancestral/wild-type virus as the prespecified homologous parent-trial endpoint. Although the analyzed Day 0-to-Day 56 sampling window ended before the nationwide Omicron wave in December 2022, ancestral-strain titers do not directly measure Omicron-lineage neutralization. Fourth, the proteomic analysis was constrained by the Olink Explore 3072 panel, and key network proteins require targeted validation (30). Although both study groups and both visits were represented on all three reconstructed Olink plates, plate was not included as a model covariate and no formal plate-association test was performed; residual plate or batch effects therefore cannot be excluded. Finally, the modest sample size, residual age and BMI imbalance, and high-dimensional nature of the omics analyses indicate that these findings should be regarded as exploratory and hypothesis-generating, requiring replication in larger independently powered cohorts. Despite these limitations, the measured proteomic and transcriptomic datasets consistently showed smaller Day 0-to-Day 56 molecular changes in the PTB group than in healthy controls. The CIBERSORT-derived cell-fraction estimates and nominal antibody–protein association patterns provide additional exploratory observations but require independent validation.

## 5 Conclusion

In conclusion, this multi-omics study shows that PTB patients attained neutralizing antibody titers comparable to healthy controls after inactivated COVID-19 vaccination, yet they exhibited attenuated and distinct systems-level molecular remodeling. The main systems-level features included baseline differences in pathways related to antigen processing and vesicle trafficking, limited proteomic and transcriptomic remodeling after vaccination, exploratory antibody-protein correlation patterns linked to inflammatory regulation, and limited computationally inferred immune-cell remodeling. These results indicate that the measured Day 56 neutralizing-antibody endpoint did not capture all between-group differences observed in the proteomic, transcriptomic, and computational deconvolution analyses. The functional and clinical relevance of these systems-level differences remains to be determined. Our findings support the need for larger longitudinal studies integrating multi-omics with direct cellular and functional immune assays to clarify the quality, durability, and clinical relevance of vaccine-induced immunity in PTB patients.

## Data Availability

The participant-level datasets presented in this article are not readily available for unrestricted public access because they were generated from a clinical trial and include human transcriptomic data linked to clinical information, which are subject to ethical, privacy, and institutional data-governance restrictions. Access to de-identified data may be considered through an institutional data transfer agreement, provided that the proposed use has appropriate ethical approval and is consistent with the participants’ informed consent. Requests to access the datasets should be directed to jingxin42102209@126.com.
