# Supplementary material for: Dissociation between humoral and multi-omic immune responses to inactivated COVID-19 vaccination in patients with pulmonary tuberculosis
Source: Front Immunol. 2026 Sep 18;17:1933038. doi: 10.3389/fimmu.2026.1933038 (PMC13631080; doi:10.3389/fimmu.2026.1933038)
Supplement: Supplementary file 1 [file DataSheet1.docx]

Figure S1. Baseline functional landscape of plasma proteomic and transcriptomic differences between PTB and healthy controls.

(a) Gene Ontology and KEGG pathway enrichment analyses of baseline plasma proteins downregulated in PTB patients relative to healthy controls. (b) Gene Ontology and KEGG pathway enrichment analyses of baseline differentially expressed genes between PTB patients and healthy controls. (c) GSEA showing pathways negatively enriched in PTB patients relative to healthy controls at baseline. P values were adjusted for multiple testing using the FDR method.

Figure S2. Baseline inferred immune cell composition differences between PTB and healthy controls.

Immune-cell fractions were estimated from PBMC transcriptomic profiles using CIBERSORT with the LM22 signature matrix. Boxplots show estimated immune-cell proportions at Day 0. Between-group comparisons were corrected for multiple testing using the FDR method. PTB, pulmonary tuberculosis; HC, healthy controls; ns, not significant.

Figure S3. GSEA of vaccination-induced transcriptomic pathway changes in healthy controls.

(a) Pathways negatively enriched at Day 56 compared with Day 0 in healthy controls. (b) Pathways positively enriched at Day 56 compared with Day 0 in healthy controls. Gene-level rankings were generated from longitudinal differential expression analysis. Pathways with FDR-adjusted P < 0.05 were considered significant. HC n=20.

Figure S4. Distribution of vaccination-induced transcriptomic fold changes in healthy controls and PTB patients.

(a) Density distribution of gene-level log2 fold changes from Day 0 to Day 56 in healthy controls and PTB patients. (b) Violin plot comparing the distribution of gene-level log2 fold changes between the two groups. Log2 fold changes were calculated as Day 56 versus Day 0 within each group based on paired PBMC RNA-sequencing data. Transcriptomic cohort: PTB n = 20, HC n = 20. PTB, pulmonary tuberculosis; HC, healthy controls; PBMC, peripheral blood mononuclear cell.

Figure S5. Inferred immune cell composition changes after vaccination within healthy controls.

Immune-cell fractions were estimated from PBMC transcriptomic profiles using CIBERSORT with the LM22 signature matrix. Boxplots show paired changes from Day 0 to Day 56 in healthy controls. P values were corrected for multiple comparisons using the FDR method. HC n = 20; ns, not significant.

Figure S6. Inferred immune cell composition changes after vaccination within PTB participants.

Immune-cell fractions were estimated from PBMC transcriptomic profiles using CIBERSORT with the LM22 signature matrix. Boxplots show paired changes from Day 0 to Day 56 in PTB patients. No immune-cell subset showed a significant change after FDR correction. PTB n=20; ns, not significant.

Figure S7. Differential inferred immune cell fractions between PTB and healthy controls after vaccination (day 56).

Immune-cell fractions were estimated from PBMC transcriptomic profiles using CIBERSORT with the LM22 signature matrix. Boxplots show estimated immune-cell proportions 28 days after the second vaccine dose. For PTB patients, Day 56 samples were collected before administration of the scheduled third dose. Between-group comparisons were corrected for multiple testing using the FDR method. PTB n=20; HC n=20; ns, not significant.
